# A Rapid and Sensitive Diagnostic Screening Assay for Detection of Mycobacteria Including *Mycobacterium tuberculosis* Directly from Sputum without Extraction

**DOI:** 10.1155/2015/593745

**Published:** 2015-01-06

**Authors:** Lisa Jane Cross, Catherine Anscombe, Timothy D. McHugh, Ibrahim Abubakar, Robert John Shorten, Nicola Thorne, Cath Arnold

**Affiliations:** ^1^Genomic Services & Development Unit, Public Health England, Microbiology Services Operations, 61 Colindale Avenue, London NW9 5HT, UK; ^2^Genomic Research Unit, Public Health England, Microbiology Services, 61 Colindale Avenue, London NW9 5HT, UK; ^3^Centre for Clinical Microbiology, Royal Free Campus, UCL and Department of Medical Microbiology, Royal Free Hampstead NHS Trust, Royal Free Hospital, Pond Street, London NW3 2PF, UK; ^4^Centre for Infectious Disease Epidemiology, Mortimer Market Centre, UCL and TB Section, Public Health England, London WC1E 6JB, UK

## Abstract

We report a novel approach utilising a real-time PCR screening assay targeting a 53 bp tandemly repeated element present at various loci within the *Mycobacterium tuberculosis (Mtb)* genome. Positive samples were identified within a discriminatory melting curve range of 90–94°C, with results obtained in under one hour directly from decontaminated sputum samples without extraction. A panel of 89 smear-positive sputa were used for analytical validation of the assay with 100% concordance, with sensitivity matching that of culture. Cross reactivity was detected within a narrow range of mycobacteria other than tuberculosis (MOTT) (five sputa, three *in silico*), with the highest sensitivity within *M. avium* complex (MAC). A year-long head to head evaluation of the test with the GeneXpert platform was carried out with 104 consecutive samples at the Royal Free Hospital, UK. Receiver operating characteristics (ROC) analysis of the data revealed that the two tests are approximately equivalent in sensitivity, with the area under the curve being 0.85 and 0.80 for the GeneXpert and our assay, respectively, indicating that the test would be a cost effective screen prior to GeneXpert testing.

## 1. Introduction

*Mycobacterium tuberculosis* (*Mtb*), the aetiological agent of tuberculosis (TB), is the second leading cause of death by infectious disease worldwide with latent infection affecting as much as one third of the world's population [1]. Rapid diagnosis is essential if treatment is to be initiated and the patient actively engaged in the treatment process.

In order to detect acid fast bacilli (AFB) in sputum, including* Mtb*, by Ziehl Neelsen staining, over 5,000 organisms per mL sputum are needed to visualise the bacilli by light microscopy [2]. As such the smear test often lacks sensitivity and specificity for detection of* Mtb* and in patients with active pulmonary TB, only an estimated 45% of infections are detected by sputum microscopy [3]. Culture of* Mtb* remains the gold standard for both diagnosis and drug sensitivity testing and can detect as low as one bacterium per mL of sputum [4]; however the technique is hampered by both long incubation times (up to several weeks for diagnosis in solid culture) and by being difficult to implement in the field [2]. It is imperative that new diagnostic methods are developed, preferably with a point-of-care (POC) technique that would be easily transferred to resource poor settings. The test must be a rapid, simple, specific, and highly sensitive method to detect* Mtb* in sputum and respiratory specimens at levels as low as a single* Mtb* genome copy.

Current molecular methodologies for* Mtb* detection and genotyping include the amplification of the transposable element IS*6110* present in* Mtb* and other members of the TB complex (MTBC) [5]. The copy number of IS*6110* in the* Mtb* genome is strain dependant, with up to 25 copies present and an average of approximately 15 copies. Some* Mtb* strains possess a single copy or lack IS*6110* entirely, thus potentially lowering assay sensitivity [6].

Simultaneous detection as well as strain differentiation of* Mtb* was reported using a technique termed “spacer oligonucleotide typing” or “spoligotyping” based on amplification of polymorphic “spacer” regions within the direct repeat (DR) locus present exclusively in MTBC strains [7]. Strains vary in the presence or absence of particular spacer regions. Unlike IS*6110*, spoligotyping is able to distinguish between* M. bovis* and* Mtb* [8].

Amplification-based TB detection kits have been developed, for example, the Roche COBAS AMPLICOR MTBC System and the BD ProbeTec ET System (BD Biosciences), but they are of inferior sensitivity compared to culture-based tests [9, 10].

Most recently the GeneXpert test from Cepheid [11, 12] has quickly become the most widespread molecular test for diagnosing tuberculosis and simultaneously detecting rifampicin resistance as indicated by mutations in the* rpo*B gene and used as a surrogate for multidrug resistance. Although sensitive, specific, and easy to use, the test and equipment are expensive and the GeneXpert platform requires a stable power source limiting its value in the poor resource countries that need it the most. A less expensive test would have clear advantages.

**Figure 1:**
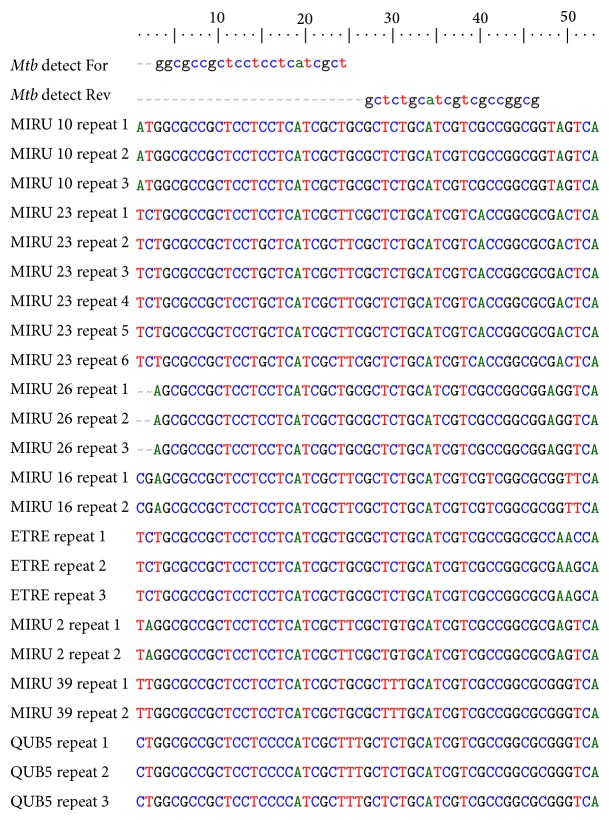
Alignment of loci containing the targeted 53 bp repeat element within a fully sequenced* M. tuberculosis* genome (H37Rv) and primers used in the study (*Mtb* detect For and* Mtb* detect Rev). Sequence labels correspond to the published name of the locus and the repeat number at that locus.

The most recent molecular marker to be widely applied for the differentiation of strains of* Mtb* is Variable Number Tandem Repeat (VNTR), also termed Mycobacterial Interspersed Repetitive Unit (MIRU) [13, 14]. The novel assay described here (hereafter referred to as “*Mtb* detect”) targets some of these tandemly repeated DNA. Varying copy numbers of the 53 bp sequence shown (usually three to 13 copies at each locus, Figure 1) are observed in the genomic DNA from a narrow range of mycobacteria including the MTBC at high copy number (30+ copies),* M. avium* complex (MAC, predicted 12–14 copies), and at single or low copy number (predicted 1–5 copies) in three other mycobacteria species (*M. marinum, M. ulcerans,* and* M. leprae)*. Here we describe the development and evaluation of an assay (*Mtb* detect) that allows single molecule detection of this highly repeated DNA element directly and inexpensively from decontaminated sputum without extraction to be used as an inexpensive screen prior to GeneXpert testing. This invention has been described in a pending International patent application, published as WO 2009/125228.

## 2. Material and Methods

### 2.1. *Mtb* Detect Test Development

#### 2.1.1. Strains for Test Development

Two* Mtb* strains, H37Rv and an in-house culture designated 5480 from a clinical isolate collected in the UK in 2010, were used as positive controls for assay development. These were cultured onto Lowenstein-Jensen slopes and identified by phenotypic and biochemical tests [15]. DNA was extracted using a QIAamp DNA mini kit (Qiagen, UK) according to manufacturer's recommendations.

#### 2.1.2. Sputum Samples for Test Development

Seventy-six smear-positive sputum samples, of which four were confirmed by culture as MOTT including* M. chelonae, M. fortuitum, M. intracellulare,* and* M. abscessus,* were obtained from the Royal London Hospital, UK. Samples were inactivated by boiling in 1 M NaOH for 10 min, following dithiothreitol liquefaction (Sputasol, Oxoid, UK). A further panel of seven inactivated sputum samples containing* Mtb* at a concentration of >90 bacilli per microscopy field and a further six sputa containing MOTT including* M. malmoense* and* M. chelonae* were obtained from the Regional Mycobacteriology Laboratory, Newcastle, UK. Decontaminated sputum samples were heat inactivated by boiling at 105°C for 10 min. Samples were stored at −20°C.

#### 2.1.3. Acid Fast Smear Microscopy Scores

All cell count calculations are based on the addition of 10 *μ*L sputum and 300 fields per slide. A result of <10 indicates an extrapolated bacterial count per sample of 1–9 on the whole slide, 1+ indicated ≥10 bacteria on the whole slide but less than 2 per field, 2+ indicates 2-3 bacteria per field, and 3+ indicates ≥3 bacteria per field.

### 2.2. Amplification

The following primers targeting the 53 bp repeat element (Figure 1) were designed and synthesised:* Mtb* detect For: 5′ GGC GCC GCT CCT CCT CAT CGC T 3′ and* Mtb* detect Rev: 5′ CGC CGG CGA CGA TGC AGA GC 3′, used for amplification on block-based, real-time, and* in silico* platforms as follows.

#### 2.2.1. Block-Based Amplification

PCRs consisted of 25 *μ*L 2x ReadyMix (Sigma, UK), 5 pmol each primer, approximately 50 ng template DNA or 1 *μ*L of inactivated sputum, and nuclease-free water to total reaction volume of 50 *μ*L. PCR cycling parameters on an Applied Biosystems 9700 thermal cycler were as follows: 95°C for 12 min, 45 cycles of 94°C for 30 sec, 64°C for 1 min, and 72°C for 2 min.

#### 2.2.2. Real-Time PCR Amplification

PCR reaction mixtures consisted of 10 *μ*L 2x Lightcycler 480 (LC480) SYBR green I Mastermix (Roche, Applied Science UK), containing FastStart Taq DNA Polymerase, reaction buffer, dNTP mix (with dUTP instead of dTTP), SYBR Green I dye and MgCl_2_ together with 0.5 *μ*M each primer, 1 *μ*L of template DNA, and nuclease-free water to 20 *μ*L. PCR cycling parameters on the Roche LC480 were as follows: 40°C Uracil-DNA N-Glycosylase (UNG) activation was carried out for 10 min by addition of 0.25 U enzyme to each reaction to digest any dUTP containing DNA present prior to cycling to prevent carryover of previously amplified samples. This was followed by denaturation at 95°C for 12 min, 45 cycles of 95°C for 10 sec, and 72°C for 1 sec. The ramping rates were 4.4 and 1.0°C/s, respectively. A melt curve protocol directly followed: 99°C for 10 sec, 55°C for 20 sec, and finally reheating to 99°C with 5 data acquisitions per °C. The ramping rates were 4.4 and 2.2°C/s, respectively.

Samples suspected to contain inhibitors (positive by smear microscopy, negative by block-based PCR) were diluted 1 : 50 in nuclease-free water prior to amplification.

#### 2.2.3. *In Silico* Amplification

The software “*in silico* Simulation of Molecular Biology Experiments” was used to estimate the specificity of the* Mtb* detect assay using the “*in silico* PCR amplification” software located at http://insilico.ehu.es/PCR/index.php?mo=Mycobacterium. Forward and reverse primer sequences were submitted and the search criteria of allowing two mismatches (none on the 3′ end) stipulated. Analysis was undertaken against all available full mycobacterial genomes (*n* = 43).

### 2.3. Analysis

#### 2.3.1. Block-Based Analysis

PCR products were sized using agarose gel electrophoresis.

#### 2.3.2. Real-Time Analysis

Amplification curves were analysed by the Absolute Quantification/2nd Derivative Max method derived from the LC480 software (Roche, UK). Melt curve analysis was automatically performed using the negative first derivative (-dF/dT) method within the LC480 software.

### 2.4. Restriction Endonuclease Digestion

Block-based generated PCR products were confirmed as being repeat-based by restriction digestion with* Hha*I according to manufacturer's instructions (New England Biolabs, UK) and visualised on an agarose gel.

### 2.5. Clinical Evaluation and Analysis

One hundred and four consecutive clinical samples of different sample type (see Table 1) were decontaminated according to commonly used practice. Briefly, samples were decontaminated with an equal volume of 4% NaOH and then neutralised followed by dithiothreitol liquefaction (Sputasol, Oxoid, UK) and centrifuged. Ten millilitres of sterile water was added and centrifuged again. An auramine stain was performed on the deposit followed by microscopy. This deposit (approximately 2.5 mL) was also used for both PCR and incubation in the BD MGIT (Becton Dickinson). Five hundred microlitres was combined with 1.5 mL GeneXpert sample reagent and incubated for 15 minutes according to manufacturer's instructions (Cepheid, USA). One microlitre of this mixture was then used as template for the* Mtb* detect assay and amplified as described above. The remaining mixture was loaded onto the instrument cartridge and processed according to manufacturer's instructions.

The diagnostic accuracy of the PCR and GeneXpert test was compared using summary receiver operator characteristic (ROC) curve analysis with culture and a clinical decision to treat each patient as gold standard in two separate analyses. All analyses were done in the statistical software Stata.

## 3. Results

BLAST searches using default settings with the* Mtb* detect primer set revealed identity to MTBC,* M. avium* complex (MAC),* M. ulcerans*,* M. marinum,* and* M. leprae*.

### 3.1. Test development: MTBC

**Figure 2:**
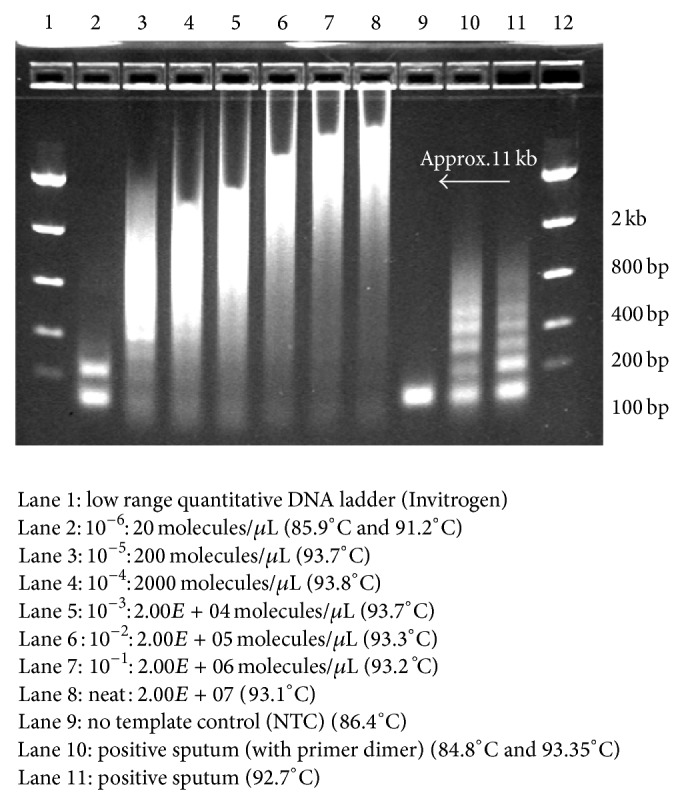
Agarose gel electrophoresis of amplified products from serial dilutions of a known concentration of* Mtb* DNA (H37Rv).

A total of 79 microscopy AFB smear-positive MTBC sputum samples were subjected to the block-based* Mtb* detect assay and analysed by agarose gel electrophoresis. Initial sample concentrations were determined by standard smear microscopy and total bacterial counts were extrapolated from sample volumes used. All negative PCR control reactions formed a distinct primer dimer of 38 base pairs (Figure 2). Samples with low level starting template (<20 molecules/*μ*L) were visualised as distinct bands (Figure 2). Samples with a higher concentration of template (~2 × 10^2^ to 2 × 10^7^ molecules/*μ*L) produced a smear ranging from 200 to 12 kbp (Figure 2).

Fifty-eight MTBC samples tested positive and 21 negative by block-based PCR and gel electrophoresis. Samples that failed to amplify were assumed to contain endogenous PCR inhibitors and were subsequently analysed using a real-time platform to increase the detection sensitivity.

**Figure 3:**
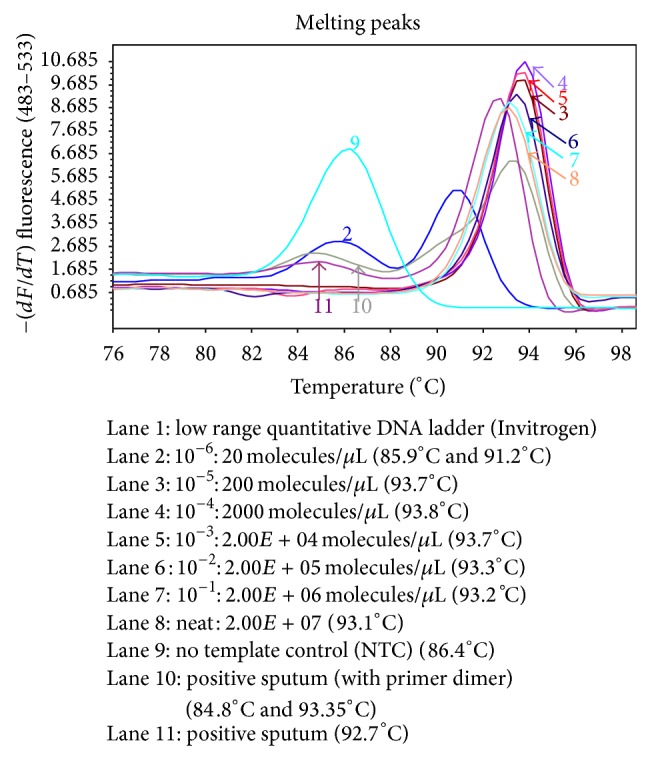
Melting curve analysis of LC480 amplified serial dilutions of H37Rv DNA.

Twenty-one smear positive MTBC sputum samples negative by the block-based method were transferred to the real-time platform. Twenty of the 21 sputa yielded a positive result on the LC480 system as judged by a melting point of 90°C–94°C. Negative controls formed primer dimers with a melting point of 86°C; low positive samples formed both products (Figure 3).

All nine samples classified as low positives (<10 bacilli/slide) tested positive by the* Mtb* detect real-time assay (after diluting 1 : 50 to decrease the effect of endogenous PCR inhibitors). Based on extrapolation of microscopy scores to bacterial counts in the original sputum sample, the real-time assay was calculated to be approximately 2–50 times more sensitive than smear microscopy and indicated lower than single cell sensitivity.

### 3.2. Test Development

Ten of the sputum samples contained MOTT. To empirically test the specificity of* Mtb* detect for the MTBC these samples were subjected to the real-time assay. Cross reactivity was shown with an* M. avium* complex (MAC) strain and with* M. chelonae, M. abscessus, M. fortuitum,* and* M. intracellulare*.

Restriction digestion was used to characterise the high molecular weight smear observed.* Hha*I with the cutting site GCGC cleaved the 44 bp repeat amplicon, theoretically producing digested products of three, 20 and 21 bp, and was used to discriminate against the primer dimer product which produced digested products of 3 and 35 bp (data not shown). All amplicons were fully cleaved, observed as disappearance of the smear and the 44 bp fragment, indicating that the high molecular weight product was exclusively composed of concatemers of the repeat element.

The specificity of the* Mtb* detect assay was tested* in silico* with 43 available sequenced mycobacterial genomes. The highest number of repeats identified by the assay was for species of the MTBC (30+ copies) except for some cross reactivity with the MAC (12–14 copies),* M. ulcerans* (four copies),* M. marinum* (five copies), and* M. leprae* (three copies).

### 3.3. Clinical Head to Head Evaluation

**Figure 4:**
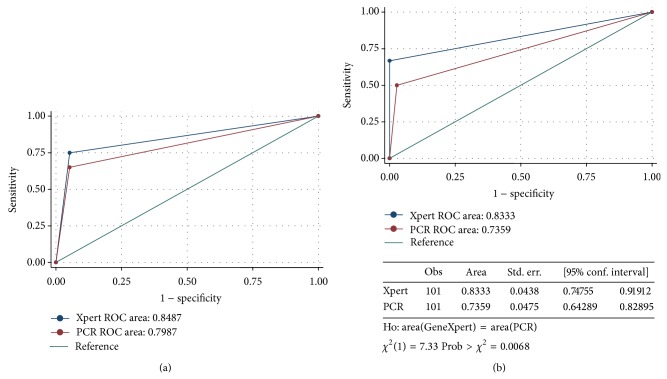
(a) Receiver operator characteristic curve for* Mtb* detect and GeneXpert compared to culture as gold standard. (b) Receiver operator characteristic curve for* Mtb* detect and GeneXpert compared to treatment as gold standard.

Table 1 shows the results of the head to head evaluation with the GeneXpert for the 26/104 samples giving a positive result for at least one of the four tests (smear, culture, GeneXpert, or* Mtb* detect PCR). The remaining 78/104 were negative by all four tests. ROC analysis of the data using bacterial culture as the gold standard, indicated that the area under the curve for GeneXpert was 0.85 (95% confidence interval 0.75–0.95) versus area under the curve for PCR 0.80 (95% confidence interval 0.69–0.91), *P* = 0.175.  Figure 4(a) suggests that the two tests are approximately equivalent. Using a clinical decision to treat as the gold standard, the area under the curve for GeneXpert was 0.83 (95% confidence interval 0.75–0.92) versus areas under the curve for PCR 0.74 (95% confidence interval 0.64–0.83), *P* = 0.175 (Figure 4(b)). No cross reactivity was seen with MOTT (*M. kansasii* and* M. chelonae*) during the evaluation.* Mtb* detect, like the GeneXpert test, was able to detect smear negative samples. The longest number of days to culture positivity in liquid culture (MGIT) where the sample was smear negative and both GeneXpert and novel assay were positive was 23 days. Overall positive predictive value versus clinical treatment was 100% and 88% for GeneXpert and our assay, and negative predictive value was 80% and 83%.

## 4. Discussion

There is a clear need for POC diagnostic tests for TB in resource-poor settings. Although sensitive, culture of mycobacteria for diagnosis of* Mtb* is not specific and additional time as well as testing is required to confirm the identification of* Mtb*, often following several weeks of culture. Culture facilities require dedicated staff with specialised training and a highly developed infrastructure making it unsuitable for use in POC settings. Smear microscopy is currently the most widely used diagnostic test for TB and is probably the most rapid but has several limitations that prevent it from being used at POC. Smear microscopy lacks sensitivity and specificity and also requires sophisticated laboratory infrastructure not available in POC settings. An ideal molecular POC test should be as sensitive as culture, highly reproducible, rapid, robust, and simple, with low biosafety and contamination risk, and should not require specialised, potentially expensive equipment such as centrifuges. Many DNA targets in the* Mtb* genome have been the focus of PCR development for rapid POC assays. Although not regarded as being as sensitive as culture, the most widely used diagnostic PCR test for* Mtb* targets the IS*6110* element which is present in multiple copies (up to 25 copies) in the genome [16]. Strains with low or no copies will result in loss of, or considerably reduced, assay sensitivity. To address this, a simple real-time PCR assay was developed that targets a tandemly repeated element present in multiple copies (approximately 20–30) in all lineages of* Mtb* [17]. The use of a Uracil-N DNA-Glycosylase incubation at the beginning of the dUTP-containing PCR ensured previously amplified products could not act as template and cross contaminate.

During analytical validation, nine weakly smear positive samples (1–9 bacilli per slide) were positive by block-based analysis and when analysed using the real-time PCR required dilution before testing positive. This is likely due to sample inhibition and should be considered when negative results are obtained [18]. Apart from low target concentration in sputum samples this inhibition may contribute to the generally observed lack of sensitivity of PCR for* Mtb*.

Although targeting the multiple repeats mainly found in MTBC, the assay also amplified the low copy numbers of these repeats identified in a restricted range of MOTT during assay development. Of the closely related MOTT that cross reacted (MAC,* M. intracellulare, M. marinum, M. malmoense,* and* M*.* ulcerans*), MAC showed cross reactivity (7/10 replicates positive) and the remainder also demonstrated cross reactivity (1/10 replicates positive). In summation the main limitation of the described assay is the cross reactivity to a number of (<10) MOTT and further clinical validation with a wider range of mycobacterial species needs to be carried out before conclusions can be drawn regarding detailed specificity information.

The head to head clinical evaluation ROC analysis of the data suggested that the two tests are approximately equivalent in sensitivity and if the bias towards the GeneXpert test (as the gold standard) is taken into account, then the novel* Mtb* detect assay may offer increased sensitivity. The area under the curve for the GeneXpert and the* Mtb* detect were 0.85 and 0.80, respectively. Specificity was equal with each assay cross reacting with* M. triviale*. Some inconsistent results were seen between methods for some samples (1, 4, 5, 16, 10, 12, 13, 19, 23, 24). For sample 1 both culture and smear result were negative but GeneXpert and* Mtb* detect were positive indicating that there were no viable organisms present but bacterial DNA was detected by the molecular tests as a second subsequent sample grew fully resistant* Mtb* (data not shown). For samples 4 and 16 all results were positive but the reference laboratory identification was for* Mycobacterium* species not* Mtb* specifically. As the GeneXpert has been shown to be specific for* Mtb* this result could mean that a mixture of mycobacteria was present, including* Mtb*, or that the laboratory identification was wrong. For sample 5 both smear and culture results were positive but molecular tests were negative, indicating that the identification of* Mycobacterium* species (but not* Mtb*) by the reference laboratory was correct. For sample 10 a GeneXpert test was positive and* Mtb* detect was negative but no other tests were done. For samples 12 and 13, all tests were negative except for* Mtb* detect. It is possible that these false positives (based on GeneXpert result) are because the* Mtb* detect is more sensitive but relies on the likelihood of a target molecule being present in the sample and due to the low input volume this is variable. It is also possible that the test is picking up low level DNA from nonviable organisms present in the sample. Contamination with amplified DNA carried over from previous tests is an unlikely scenario as UNG is employed prior to amplification to digest amplified DNA. For sample 19 a GeneXpert test was positive and* Mtb* detect was negative with a negative smear and culture result. No follow-up results were available for this sample. The false negative from the* Mtb* detect test in this case was most likely due to stochastic events because of the small input volume (1 *μ*l compared to 500 *μ*l in the GeneXpert). Sample 23 was smear positive but culture negative with a positive result from both GeneXpert and* Mtb* detect, indicating that the sample contained only killed mycobacteria or that there was a mix-up with culture. Sample 24 also gave a smear positive but culture negative result but was positive using GeneXpert but negative with* Mtb* detect, again possibly because of low input volume.

Cross reactivity was observed with both the GeneXpert and* Mtb* detect. Analysis of the* rpo*B sequence of* M. triviale* (data not shown) indicates that this cross reactivity should not be possible and is therefore more likely to be either a mixed sample or an incorrect identification from the reference laboratory.

Further development is required to either enable concentration of the target DNA from the crude extract (possibly by bead based capture to fit with POC principles) or remove inhibitors or a combination of both to enable the addition of a larger input volume in the* Mtb* detect test to determine whether the stochastic behaviour of low concentration molecules in a small volume is the reason behind the false positive/false negative results of the test compared to the GeneXpert.

However, the current value of the test is as an effective screen with confirmation of positives by GeneXpert testing which also provides an indication of rifampicin resistance as an early indicator of multidrug resistance. Work is ongoing to address the slightly lower specificity indicated during analytical validation of* Mtb* detect to include an additional* Mtb* specific marker(s).

The cartridge-based GeneXpert assay is highly sensitive and specific; however it is not feasible to implement it in rudimentary POC settings due to cost and infrastructure limitations; it requires dedicated personnel and is not portable; despite roll-out backed by WHO it has made little impact outside the Republic of South Africa. The use of a simple, cost effective, and sensitive screen with the potential to be used at POC will, in the short term, reduce the cost of GeneXpert testing considerably and with refinement may provide a viable alternative to GeneXpert (with 500-fold input volume reduction and direct sample processing).

The assay can be performed and read by nonspecialist personnel and is non-labour intensive. The cost per sample is approximately *£*0.45 at the time of writing and can be scaled up to 384-well throughput.

## 5. Conclusions

A highly sensitive, cross-platform, diagnostic screening assay for the detection of* Mtb,* together with a narrow range of MOTT, directly from decontaminated sputum was developed without a time-consuming nucleic acid extraction procedure making it more suitable for adaptation to POC use and with a turnaround time of around one hour.

## Figures and Tables

**Table 1:** *Mtb* detection and GeneXpert head to head clinical evaluation results.

Sample number	Sample type	Smear result	Culture	GeneXpert result	*Mtb* detection result	Days to positivity	Reference lab ID
1	Sputum	Neg	Neg	**Pos**	**Pos**		
2	Sputum	**Pos**	**Pos**	**Pos**	**Pos**	6	*M.tuberculosis *
3	Sputum	**Pos**	**Pos**	**Pos**	**Pos**	5	*M.tuberculosis *
4	Sputum	**Pos**	**Pos**	**Pos**	**Pos**	4	*Mycobacterium *sp.
5	Sputum	**Pos**	**Pos**	Neg	Neg	11	*Mycobacterium *sp.
6	Sputum	Neg	Pos	Neg	Neg	8	*M.kansasii *
7	Sputum	**Pos**	**Pos**	Neg	Neg	5	*M.kansasii *
8	Sputum	**Pos**	**Pos**	Neg	Neg	5	*M.kansasii *
9	Sputum	Neg	**Pos**	**Pos**	**Pos**	14	*M.tuberculosis *
10	Sputum	ND	ND	**Pos**	Neg		
11	Sputum	**Pos**	**Pos**	**Pos**	**Pos**	4	*M.tuberculosis *
12	Bronchial washing	Neg	Neg	Neg	**Pos**		
13	Bronchial washing	Neg	Neg	Neg	**Pos**		
14	Bronchial washing	Neg	**Pos**	**Pos**	**Pos**	8	*M.tuberculosis *
15	Bronchial washing	Neg	**Pos**	**Pos**	**Pos**	10	*M.tuberculosis *
16	Bronchial washing	**Pos**	**Pos**	**Pos**	**Pos**	10	*Mycobacterium *sp.
17	Bronchial washing	**Pos**	**Pos**	**Pos**	Neg	7	*M.tuberculosis *
18	Bronchial washing	Neg	**Pos**	Neg	Neg	12	*M.chelonae *
19	Biopsy	Neg	Neg	**Pos**	Neg		
20	Biopsy	**Pos**	**Pos**	**Pos**	Neg	23	
21	Fluid	Neg	**Pos**	**Pos**	**Pos**	23	*M.tuberculosis *
22	Fluid	**Pos**	**Pos**	**Pos**	**Pos**	14	*M.triviale *
23	Fluid	**Pos**	Neg	**Pos**	**Pos**		
24	Fluid	**Pos**	Neg	**Pos**	Neg		
25	Fluid	**Pos**	**Pos**	**Pos**	**Pos**	4	*M.tuberculosis *
26	Pus	**Pos**	**Pos**	**Pos**	**Pos**		*M.tuberculosis *

ND = not done.
